# Accident prevention in radiotherapy

**DOI:** 10.2349/biij.3.2.e27

**Published:** 2007-04-01

**Authors:** O Holmberg

**Affiliations:** Physics Department, Copenhagen University Hospital, Herlev, Herlev Ringvej 75, 2730 Herlev, Denmark

**Keywords:** Risk management, quality assurance, accident, incident, error

## Abstract

In order to prevent accidents in radiotherapy, it is important to learn from accidents that have occurred previously. Lessons learned from a number of accidents are summarised and underlying patterns are looked for in this paper. Accidents can be prevented by applying several safety layers of preventive actions. Categories of these preventive actions are discussed together with specific actions belonging to each category of safety layer.

## INTRODUCTION

Preparation and execution of radiotherapeutic treatment is a complex task with many inherent hazards. When considering the potential risks in radiotherapy, it should, however, always be recognised that the treatment has a potential substantial benefit to the patient.

In attempting to avoid accidents in radiotherapy, it is very important to remember the lessons that can be learned from previous radiotherapy accidents and to ensure that preventive actions are applied in a clinical setting. A number of accidents have been thoroughly investigated and the lessons learned have been disseminated [1-4] by the International Atomic Energy Agency (IAEA). The International Commission on Radiological Protection (ICRP) has summarised causes and contributory factors for radiotherapy accidents in 2000 [5].

Prevention of accidents in radiotherapy involves applying several layers of preventive actions, addressing this issue at several levels. It is suggested [6] that these layers encompass:

1. Actions where potential deviations from intended dose and geometry can be found before the first irradiation-fraction of the patient;
2. Actions where deviations can be found during or after the treatment course;
3. Application of safety-technology;
4. Application of safety procedures; and
5. Actions where contributing factors such as staffing-levels and structure, training and communication are addressed.

The first objective of this review is to assess common aspects of lessons learned from major radiotherapy accidents in order to highlight patterns seen during accidents. This follows a review performed by the author of the creation of an IAEA regional training course on prevention of accidental exposure in radiotherapy. The second objective is to identify actions within the preventive layers as suggested above.

## LESSONS LEARNED FROM MAJOR RADIOTHERAPY ACCIDENTS

Specific lessons learned from some of the major radiotherapy accidents are presented below. Case histories are not presented in detail, as they have been described in the literature. Finally, the lessons learned are grouped under four headings, highlighting patterns seen in the lessons learned.

### Incorrect decay data (USA) [7]

During a time period of two years, a physicist failed to perform regular measurements [calibrations and quality assurance (QA)] on a cobalt unit for radiotherapy but instead relied on estimations of the decay of the source in order to predict the dose rate for calculation of the treatment time. The dose rate was plotted on a graph paper and the dose rate was extrapolated over time. This extrapolation was done incorrectly, resulting in the patients receiving overdoses of 10% to 55%.

Some of the specific lessons learned from this accident were:

1. Independent check of a physicist’s work should be performed
2. Formal procedures for calibrating the treatment unit on a regular schedule should exist and be followed.
3. A department should provide sufficient staff to handle the workload.
4. Records must accurately document the performance of accepted QA procedures.

### Erroneous use of treatment planning system (UK) [8]

When a computerised treatment planning system (TPS) was brought into clinical use, a hospital began treating with isocentric techniques. The TPS correctly applied an inverse-square correction for these treatments. Not aware of this, an additional distance correction factor was applied manually by the persons calculating treatment time. A distance correction factor was thus applied twice for all patients treated isocentrically, causing patients to receive doses lower than prescribed. The incorrect procedures were found to have been in place for approximately nine years before they were discovered.

Specific lessons learned include:

1. Staff should be properly trained in the operation of the equipment and understand the operating procedures.
2. Quality Assurance Programme procedures should include complete commissioning of treatment planning equipment before first use, and procedures for independent checking of patient treatment time calculations.

### Accelerator software problems (USA and Canada) [9]

A specific type of accelerator relied on software for safety interlocks (and not, as in other models, mechanical and electrical safety interlocks). Several accidents occurred involving unintended carousel positioning prior to treatment, resulting in extremely high electron energy fluence directed towards the patient.

Some of the specific lessons learned from this accident are:

1. Patient reactions should be observed, reported and followed up, and all reports of abnormal machine operation should also be investigated.
2. The Quality Assurance Program should include a review of procedures for reporting unusual events.
3. Only the software for safety cannot be relied on.

### Computer file not updated (USA) [10]

Data for treatment time calculations was updated at the exchange of a cobalt source by a medical physicist, except data for treatment with cobalt beam trimmer bars. It was stated by the oncologist that trimmer bars would not be used for treatment anymore. Some time later, treatment with trimmer bars was initiated again. The old computer file was used for calculations, but this file contained the outdated source activity, leading to patient treatment times that were too long and produced corresponding overdoses.

Some specific lessons learned:

1. Develop procedures that clearly indicate the software commissioned for clinical use, and software that has been removed from clinical service.
2. The Quality Assurance Program should include procedures for verifying the correct function of software for patient calculations.
3. Perform manual calculations to confirm computer calculations of treatment time (and use in vivo dosimetry).

### Incorrect repair of accelerator (Spain) [11]

At the breakdown of a linear accelerator, a company technician on another mission was called to the accelerator. Repair work was started and a beam was recovered. However, a meter display indicated an energy selection problem. Treatments were allowed to resume. Due to a transistor having short-circuited, a full current was fed to the magnet system all the time, making it possible to get a beam only when maximum electron energy was used. The repair work had been incorrect, and the resulting beams led to severe patient overdoses.

Some specific lessons learned:

1. The Quality Assurance Programme should include formal procedures for returning medical equipment after maintenance, including making it mandatory to report to the Physics group, before resuming treatment with patients.
2. There should be consideration of the need to verify the radiation beam by the Physics group when the repair might have affected beam parameters.
3. There should be a procedure to perform a full review or investigation when the radiotherapy equipment has unusual displays or behaviour.

### Miscalibration of beam (Costa Rica) [1]

When a new cobalt source replaced an old one, the medical physicist made an incorrect interpretation of 0.3 minutes as being 30 seconds (as opposed to the correct interpretation of 18 seconds) during calibration measurements. Consequently, the treatment times to be used were overestimated by 66%, resulting in severe overdoses.

Some specific lessons learned:

1. Ensure there is a high level of training and competence in a clinic, to ensure safe use of potentially hazardous sources.
2. Ensure there are provisions to stimulate working with awareness (e.g., a new source is expected to require shorter treatment times).
3. Ensure there are written procedures for calibration of beams and for independent verification of safety critical tasks before clinical implementation.

### Error in TPS data entry (Panama) [2]

The TPS used in a clinic had limitations in the calculations and presentation of results. To overcome these limitations, a new way of entering data was devised locally. The TPS accepted this new data entry, without giving a warning, but calculated incorrect treatment times. The result was severe overdoses to several patients.

Some specific lessons learned:

1. Manufacturers should avoid ambiguity in instructions and perform thorough testing of software, also for non-intended use.
2. The TPS is a safety critical piece of equipment.
3. Quality control should include TPS and a change in procedures should be validated before being put into clinical use.
4. Computer calculation should be verified, at least through manual checks for one point.
5. Awareness of staff for unusual treatment parameters should be stimulated and trained.

### Accelerator interlock failure (Poland) [3]

After a power failure involving a clinic, an accelerator was automatically shut down. At restoration of electrical power, the accelerator was restarted. Some tests were completed, indicating a low dose rate, leading to the filament current limitation being increased to a high level by staff so that the remaining treatments could be completed. Unfortunately, there had been a double fault: firstly a fault in a fuse of the power supply to the beam monitoring system, leading to a high dose rate, and secondly a diode was broken in the safety interlock chain. The combination of these faults, meant that no problem was indicated, while the dose rate was in fact many times higher than intended.

Some specific lessons learned:

1. There should be an immediate check upon power supply shutdowns or unusual display of unit, and a written procedure to ensure that this check was done.

### Patterns in the lessons learned

A report on several accidents in radiotherapy published by the IAEA [4], reviewed together with the specific lessons learned from the cases above, indicate that there are patterns in the lessons learned. It can be argued that most of the reported accidents occurred when certain conditions have been fulfilled. These conditions can be grouped as listed below:

1. Working with awareness and alertness: Accidental exposures have occurred owing to inattention to details, and lack of alertness and awareness. This could also be made worse if the personnel have to work in conditions prone to distractions.
2. Procedures: Accidental exposures have occurred when there is a lack of procedures and checks, or when they are not comprehensive, documented or fully implemented.
3. Training and understanding: Accidental exposures have occurred when there is a lack of qualified and well-trained staff, with necessary educational background and specialised training.
4. Responsibilities: Accidental exposures have occurred when there are gaps and ambiguities in the functions of personnel along the lines of authority and responsibility. In these cases, safety critical tasks have been insufficiently covered.

## PREVENTIVE MEASURES

Human errors should always be expected, leading to the conclusion that there should be defences in place. When a hazard is realised, it is due to weaknesses in this defence. These weaknesses can be seen as a combination of two factors, with the first factor being active failures (mistakes, lapses and procedural violations) and the second factor being latent conditions (i.e., conditions built into the system such as understaffing, high workload, and inadequate procedures or equipment). This approach follows Reason’s model [12]. Several layers of preventive actions should be put in place.

### Actions where potential deviations from intended dose and geometry can be found before the first irradiation-fraction of the patient

Independent verification of calculations has been seen to be lacking in several of the accidents presented above. There are indications that a recently reported accident in Glasgow [13] might have been prevented if a truly independent calculation check had been used. The independency of the check is vital to be able to find parameters that are not the same as intended. Many mistakes in the calculation process are due to mistakes in the act of transferring information. Another example of action in this safety-layer is clinical peer review of treatment preparation (e.g., dose and volume to be irradiated).

### Actions where deviations can be found during or after the treatment course

In vivo dose measurement is a way of finding deviations after one or a few treatment fractions. This is regularly performed with diodes. Systematic dose deviations as low as about 1-2% that affected large groups of patients, have been found by diode systems. Another action belonging to this safety-layer is clinical monitoring of adverse effects in patients.

### Application of safety-technology

An example of safety-technology to serve as a safety layer for the prevention of radiotherapy accidents is integrated radiotherapy networking. This implies the automatic transfer of parameters and images as well as the RV-system on linear accelerators. The most comprehensive level is the full integration of images and parameters throughout the treatment chain, without breaking the chain for manual transfer of information. However, a department often has a mix of electronic and manual parameter transfer. It should also be recognised that even if the full integration of equipment decreases the likelihood of mistakes in transfer of information, it does not necessarily remove the mistakes done in the creation of information. Video and audio monitoring of patients are more examples from this safety-layer.

### Application of safety procedures

There are many types of safety procedures to be put in place in order to increase safety in radiotherapy. One example is the utilisation of an incident reporting system. This has been successfully employed in a non-medical setting for many years, enhancing safe practice. The objective is for the organisation to learn from events within and outside the organisation. Potential incidents (near misses) are important in this context. Another example here is the use of documentation systems for procedures.

### Actions where contributing factors such as staffing-levels and structure, training and communication are addressed

Comprehensive training of all staff is mandatory. It is important that staff have a full understanding of the equipment being used as well as the data used. The department should also make sure that all responsibilities are allocated and understood, and that the members of staff they have been allocated to are educated accordingly and kept up-to-date in training.
